# Anti-N-Methyl-D-Aspartate (NMDA) Receptor Encephalitis Presenting as Acute Psychosis and Seizures in a Patient With Multiple Sclerosis: A Diagnostic Challenge

**DOI:** 10.7759/cureus.110612

**Published:** 2026-06-10

**Authors:** Alireza Izadian Bidgoli, Yaroslav Buryk, Amanda Pina

**Affiliations:** 1 General Surgery, American University of the Caribbean School of Medicine, Cupecoy, SXM; 2 Pulmonary and Critical Care, Jackson Memorial Hospital, Miami, USA; 3 Internal Medicine, Ross University School of Medicine, Bridgetown, BRB

**Keywords:** anti-nmda receptor encephalitis, autoimmune encephalitis, catatonia and seizures, multiple sclerosis flare-ups, neuropsychiatric symptoms

## Abstract

Autoimmune encephalitis (AE) is a potentially reversible cause of acute neuropsychiatric deterioration; however, diagnosis may be delayed when symptoms overlap with those of pre-existing neurologic disorders. We report the case of a 27-year-old woman with known multiple sclerosis (MS) who presented with acute psychosis and rapidly progressed to focal seizures, encephalopathy, catatonia, dyskinesias, and autonomic instability. Initial neuroimaging demonstrated no evidence of active demyelination or other acute intracranial pathology, raising diagnostic uncertainty regarding atypical MS relapse, infectious encephalitis, and other inflammatory processes. Cerebrospinal fluid (CSF) analysis revealed marked lymphocytic pleocytosis, while extensive infectious studies were negative. Electroencephalography demonstrated focal seizure activity arising from the left frontal region, followed by diffuse slowing with superimposed beta activity suggestive of a delta brush-like pattern. Given the characteristic clinical progression and inflammatory CSF findings, AE was strongly suspected, and immunotherapy with high-dose corticosteroids and plasma exchange was initiated before diagnostic confirmation. The diagnosis was ultimately established by positive anti-N-methyl-D-aspartate (NMDA) receptor antibodies in both CSF and serum. This case highlights the importance of recognizing clinical features atypical for MS relapse, including acute psychosis, seizures, and rapidly progressive encephalopathy, and underscores the risk of diagnostic anchoring in patients with established neurologic disease. Early recognition of anti-NMDA receptor encephalitis and prompt initiation of immunotherapy remain critical to optimizing outcomes in this potentially reversible condition.

## Introduction

Autoimmune encephalitis (AE) comprises a group of immune-mediated inflammatory disorders of the central nervous system characterized by rapidly progressive neuropsychiatric symptoms, including psychiatric disturbances, seizures, cognitive impairment, and altered consciousness [1,2]. Among its subtypes, anti-N-methyl-D-aspartate (NMDA) receptor encephalitis is one of the most common and best-characterized forms and is mediated by autoantibodies directed against neuronal surface NMDA receptors, resulting in receptor internalization and synaptic dysfunction [1,3]. Clinically, the disorder often follows a characteristic progression beginning with psychiatric manifestations and evolving to seizures, encephalopathy, movement disorders, and autonomic instability [2-4]. Early diagnosis remains challenging because neuroimaging is frequently normal or nonspecific, while presenting symptoms may mimic primary psychiatric disorders or infectious encephalitis [2,4,5]. Importantly, delayed recognition and treatment are associated with worse neurologic outcomes, emphasizing the need for early clinical suspicion and prompt initiation of immunotherapy [3,6].

The diagnostic challenge becomes even greater in patients with pre-existing neurologic disease such as multiple sclerosis (MS). MS is a chronic immune-mediated demyelinating disorder that typically presents with focal neurologic deficits, including sensory disturbances, motor weakness, visual symptoms, and gait dysfunction [7]. Although cognitive dysfunction and psychiatric symptoms may occur, acute psychosis, seizures, and rapidly progressive encephalopathy are uncommon manifestations of MS relapse and should prompt consideration of alternative diagnoses [7,8]. Failure to recognize this distinction may result in diagnostic anchoring, in which new symptoms are prematurely attributed to an established diagnosis despite features that fall outside its expected clinical spectrum [5,8].

We present a case of anti-NMDA receptor encephalitis in a patient with known MS who developed acute psychosis followed by seizures and progressive encephalopathy. This case highlights the importance of maintaining a broad differential diagnosis, recognizing clinical features atypical for MS relapse, and considering AE when new neuropsychiatric symptoms evolve beyond the expected pattern of a known neurologic disease.

## Case presentation

Clinical presentation

A 27-year-old woman with a history of MS and obesity status post sleeve gastrectomy (December 2025) presented on March 15, 2026, with an acute onset of auditory hallucinations, homicidal ideation, altered mental status, and behavioral disturbances consistent with psychosis. She had no known prior psychiatric history. She also reported a prior history of an ovarian cyst, raising the possibility of an underlying teratoma.

Over the following days, her neuropsychiatric symptoms progressively worsened, with the development of severe encephalopathy and persistent tachycardia. She was initially treated at an outside facility with empiric thiamine therapy for presumed Wernicke encephalopathy without clinical improvement and was subsequently transferred for further neurologic evaluation. On March 24, 2026, she experienced two focal seizures, prompting neurology consultation. The progression from isolated psychiatric symptoms to seizures and encephalopathy raised concern for an underlying central nervous system inflammatory process rather than a primary psychiatric disorder alone.

Given her underlying MS, the differential diagnosis initially included atypical MS relapse, AE, and infectious encephalitis. Diagnostic evaluation subsequently included cerebrospinal fluid (CSF) analysis, EEG, neuroimaging, and infectious studies. During hospitalization, the patient developed additional neurologic manifestations including catatonia, mutism, orofacial dyskinesias, and autonomic instability characterized by persistent tachycardia and intermittent fever. These evolving clinical features progressively increased suspicion for anti-NMDA receptor encephalitis.

Evaluation (history, physical exam, laboratory, and imaging results)

On initial evaluation, the patient was encephalopathic, intermittently agitated, and inconsistently responsive to verbal commands. Neurologic examination was limited by poor cooperation but did not demonstrate clear focal motor deficits. Vital signs were notable for intermittent fever and tachycardia, while cardiopulmonary and abdominal examinations were otherwise unrevealing.

Initial laboratory evaluation demonstrated leukocytosis with neutrophilic predominance, mild microcytic anemia, hyperglycemia, and mild transaminitis. Over the course of hospitalization, inflammatory and metabolic abnormalities partially improved, although anemia persisted. A detailed summary of laboratory findings at presentation and during hospitalization is provided in Table 1.

**Table 1:** Summary of Laboratory Findings at Admission and During Hospitalization. This table summarizes selected laboratory parameters obtained at the time of hospital admission and throughout hospitalization. Admission values represent the earliest recorded measurements upon presentation. Hospitalization ranges represent the minimum and maximum recorded values observed during the hospital course and are included to demonstrate overall laboratory trends over time. Laboratory findings demonstrated initial leukocytosis with neutrophilic predominance, persistent microcytic anemia, metabolic abnormalities including hyperglycemia and electrolyte disturbances, and mild transaminitis. Serial laboratory monitoring showed partial resolution of inflammatory and metabolic abnormalities, while hematologic abnormalities persisted throughout hospitalization, consistent with a systemic inflammatory process in the setting of autoimmune encephalitis. WBC: white blood cell count; RBC: red blood cell count; Hgb: hemoglobin; Hct: hematocrit; MCV: mean corpuscular volume; MCH: mean corpuscular hemoglobin; MCHC: mean corpuscular hemoglobin concentration; RDW: red cell distribution width; Plt: platelet count; Na: sodium; K: potassium; Cl: chloride; CO₂: bicarbonate; BUN: blood urea nitrogen; Cr: creatinine; Glu: glucose; Ca: calcium; Mg: magnesium; AST: aspartate aminotransferase; ALT: alanine aminotransferase; ALP: alkaline phosphatase; eGFR: estimated glomerular filtration rate.

Laboratory Test (Unit)	Admission Value	Observed Range During Hospitalization	Reference Range
WBC (×10³/µL)	18.0	8.1-17.9	4.0–11.0
Hemoglobin (g/dL)	11.5	9.9-10.8	12.0–16.0
Hematocrit (%)	37.0	32.6-34.9	36–46
MCV (fL)	76.8	75.5–77.5	80–100
Platelets (×10³/µL)	312	262–334	150–400
Neutrophils (%)	91.3	91.1	40–70
Glucose (mg/dL)	218	190–262	70–99
Sodium (mmol/L)	140	135–148	135–145
Potassium (mmol/L)	5.4	3.1–5.4	3.5–5.0
Chloride (mmol/L)	105	100–113	98–107
Bicarbonate (mmol/L)	23	20–26	22–29
BUN (mg/dL)	20	20	7–18
Creatinine (mg/dL)	0.60	0.50-0.80	0.6–1.3
Osmolality (mOsm/kg)	299	307	275–295
AST (U/L)	91	44-92	10–40
ALT (U/L)	104	104	7–56
Alkaline Phosphatase (U/L)	98	95-130	44–147
Magnesium (mg/dL)	2.3	1.8-2.3	1.7–2.2
Lactate (mmol/L)	1.5	1.1-1.5	0.5–2.2
D-dimer (µg/mL)	0.69	—	<0.50
Procalcitonin (ng/mL)	0.14	—	<0.10

CSF analysis demonstrated lymphocytic pleocytosis, with a white blood cell count of 145 cells/µL and 100% lymphocytic predominance, along with normal glucose and protein levels. A summary of CSF findings is provided in Table 2. These findings supported an inflammatory central nervous system process. A broad infectious evaluation, including bacterial cultures, viral polymerase chain reaction testing, respiratory viral testing, HIV, syphilis, and other serologic studies, was negative.

**Table 2:** Cerebrospinal Fluid Findings and Diagnostic Studies. Cerebrospinal fluid (CSF) analysis demonstrated marked lymphocytic pleocytosis with normal glucose and protein levels, supporting an inflammatory central nervous system process. Extensive infectious evaluation, including bacterial cultures, viral polymerase chain reaction (PCR) testing, respiratory viral testing, HIV screening, and syphilis serology, was negative. Definitive diagnosis was established by detection of anti-NMDA receptor antibodies in both CSF (titer 1:10) and serum (titer 1:40), supporting anti-NMDA receptor encephalitis. CSF: cerebrospinal fluid; WBC: white blood cell count; RBC: red blood cell count; IgG: immunoglobulin G; PCR: polymerase chain reaction; HSV: herpes simplex virus; VZV: varicella-zoster virus; HIV: human immunodeficiency virus; RPR: rapid plasma reagin.

Test	Result	Reference Range / Interpretation
CSF WBC	145 cells/µL	0–5 cells/µL
CSF RBC	110 cells/µL	0 cells/µL
CSF Glucose	61 mg/dL	40–70 mg/dL
CSF Protein	30 mg/dL	15–45 mg/dL
CSF IgG	2.50 mg/dL	0–6 mg/dL
CSF Differential	100% lymphocytes	Predominantly lymphocytic
CSF Bacterial Culture	Negative	No growth
CSF Viral PCR Panel	Negative	No viral pathogen detected
HSV PCR	Negative	Negative
VZV PCR	Negative	Negative
Respiratory Viral Panel	Negative	Negative
HIV Testing	Negative	Negative
RPR (Syphilis Testing)	Negative	Negative
Anti-NMDA Receptor Antibody (CSF)	Positive (1:10)	Abnormal
Anti-NMDA Receptor Antibody (Serum)	Positive (1:40)	Abnormal

Brain magnetic resonance imaging (MRI) with and without contrast did not demonstrate acute intracranial abnormalities, active demyelination, diffusion restriction, or abnormal enhancement. Pelvic imaging, including transvaginal ultrasound and MRI pelvis, did not identify an ovarian teratoma or other pelvic mass.

**Figure 1:**
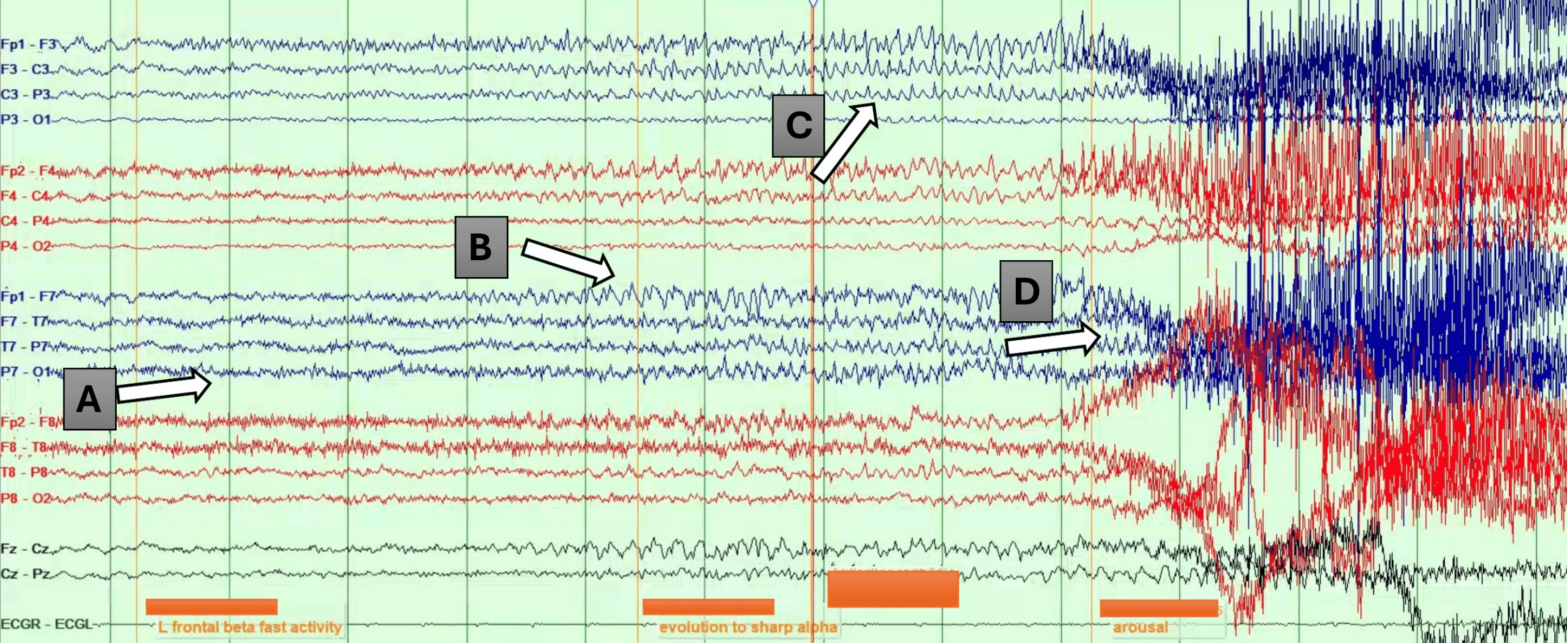
Electroencephalography (EEG) Findings Demonstrating Evolving Cortical Activity. The seizure originates in the left frontal region with rhythmic beta activity (A-B), progresses to faster rhythmic activity and sharp alpha/theta discharges (C-D), and subsequently propagates contralaterally with superimposed electromyographic artifact during the tonic-clonic phase. These findings were interpreted by the epilepsy service as consistent with a focal seizure arising from the left frontal lobe with secondary generalization.

EEG initially demonstrated focal epileptiform activity with electrographic seizures arising from the left frontal region, including both focal unaware seizures and focal-to-bilateral tonic-clonic seizures. Subsequent prolonged EEG monitoring demonstrated diffuse background slowing consistent with encephalopathy and superimposed beta activity interpreted by the treating epilepsy team as resembling an extreme delta brush pattern (Figure 1). No persistent epileptiform discharges were observed on later recordings. The EEG findings were considered supportive of anti-NMDA receptor encephalitis in the appropriate clinical context but were not considered diagnostic in isolation.

Taken together, the combination of progressive neuropsychiatric decline, seizures, lymphocytic CSF pleocytosis, negative infectious studies, nondiagnostic neuroimaging, absence of an identifiable tumor, and evolving EEG abnormalities shifted diagnostic consideration toward AE, particularly anti-NMDA receptor encephalitis.

Multidisciplinary board discussion

Given the patient's rapidly progressive neuropsychiatric deterioration and inconclusive initial diagnostic evaluation, the case was reviewed collaboratively by neurology, infectious disease, psychiatry, and critical care teams. Early in the hospitalization, the differential diagnosis remained broad and included atypical MS relapse, infectious encephalitis, primary psychiatric illness, and AE.

The patient's initial presentation with acute psychosis and behavioral disturbances was not immediately diagnostic, particularly in the setting of a known history of MS. Although neuropsychiatric manifestations may occur in MS, the subsequent development of focal seizures and progressive encephalopathy raised concern for a superimposed central nervous system process. Infectious etiologies were also strongly considered because of intermittent fever, leukocytosis, and inflammatory CSF findings. As a result, empiric antimicrobial therapy was initiated while extensive infectious testing was pursued.

As diagnostic studies became available, several findings argued against both infectious encephalitis and active MS relapse. Neuroimaging failed to demonstrate new demyelinating lesions, abnormal enhancement, or other acute structural abnormalities. Infectious studies, including bacterial cultures, viral PCR testing, and serologic investigations, were unrevealing. At the same time, the patient developed a constellation of features increasingly characteristic of anti-NMDA receptor encephalitis, including seizures, severe encephalopathy, catatonia, orofacial dyskinesias, and autonomic instability. Concurrent EEG abnormalities and lymphocytic CSF pleocytosis further strengthened suspicion for an autoimmune encephalitic process.

Following a multidisciplinary review, anti-NMDA receptor encephalitis emerged as the leading diagnostic consideration. Given the patient's continued neurologic deterioration and the recognized importance of early treatment in AE, the decision was made to initiate immunomodulatory therapy before antibody confirmation. This collaborative diagnostic and therapeutic approach facilitated timely intervention while definitive testing remained pending and ultimately proved consistent with the final diagnosis of anti-NMDA receptor encephalitis.

Management

Management required a parallel diagnostic and therapeutic approach because the patient continued to experience progressive neurologic deterioration while diagnostic evaluation remained ongoing. Initial management included seizure control with lacosamide 200 mg twice daily and valproic acid 1,500 mg twice daily, in addition to supportive care and close neurologic monitoring.

Given intermittent fever, leukocytosis, and inflammatory CSF findings, infectious encephalitis remained an important consideration early in the hospital course. Empiric antimicrobial and antiviral therapy was initiated while infectious studies were pending. Acyclovir was continued during the initial evaluation for possible viral encephalitis, and broad-spectrum antibiotics, including cefepime and vancomycin, were used during the infectious workup. These therapies were later de-escalated as infectious studies remained negative and the clinical picture increasingly favored autoimmune encephalitis.

With worsening encephalopathy, catatonia, orofacial dyskinesias, autonomic instability, and evolving electroencephalographic abnormalities, immunotherapy was initiated before antibody confirmation. The patient received high-dose intravenous methylprednisolone 1 g on non-plasma exchange days, followed by a planned oral prednisone taper beginning at 80 mg daily. Plasma exchange was initiated as first-line immunotherapy and completed for a total of five sessions. This decision was made by the multidisciplinary team, given the patient’s progressive neurologic decline and high clinical suspicion for anti-NMDA receptor encephalitis.

Definitive diagnosis was subsequently established by detection of anti-NMDA receptor antibodies in both CSF, with a titer of 1:10, and serum, with a titer of 1:40. Immunotherapy was continued after diagnostic confirmation.

Psychiatry was consulted for catatonia and neuropsychiatric manifestations. The patient was treated with scheduled lorazepam, memantine, aripiprazole, and supportive behavioral management. Autonomic instability, including intermittent tachycardia and tachypnea, was managed supportively with close monitoring and propranolol.

Given the known association between anti-NMDA receptor encephalitis and ovarian teratoma, pelvic imaging was performed and demonstrated no evidence of ovarian teratoma or other pelvic mass, supporting a nonparaneoplastic presentation.

Second-line immunotherapy was considered in the context of severe disease and incomplete early recovery. The treating team discussed enrollment in a clinical trial for anti-NMDA receptor encephalitis, including possible escalation to additional immunotherapy versus standard of care. Intravenous immunoglobulin, rituximab, or other second-line agents were not documented as administered in the available records at the time of manuscript preparation.

Clinical outcome

The diagnosis was ultimately confirmed as anti-NMDA receptor encephalitis based on positive antibody testing in both CSF and serum. The patient's clinical course was characterized by progression from acute psychosis to focal seizures, severe encephalopathy, catatonia, orofacial dyskinesias, and autonomic instability, consistent with the characteristic disease trajectory of anti-NMDA receptor encephalitis.

Following initiation of immunotherapy with high-dose corticosteroids and plasma exchange, the patient remained under close neurologic monitoring in the Neurocritical Care Unit. Seizure activity was controlled with antiepileptic therapy; however, significant encephalopathy and neuropsychiatric dysfunction persisted, necessitating ongoing multidisciplinary management involving neurology, psychiatry, infectious disease, and critical care teams.

Evaluation for an underlying paraneoplastic source, including ovarian teratoma, was negative, supporting a nonparaneoplastic presentation. At the time of manuscript preparation, the patient remained hospitalized and continued to receive active treatment and monitoring. Consequently, long-term outcome data regarding neurologic recovery, cognitive function, psychiatric recovery, discharge disposition, seizure recurrence, and maintenance immunotherapy were not yet available.

To further clarify the evolution of symptoms, diagnostic evaluation, and therapeutic interventions, a chronological summary of the patient's clinical course is provided in Table 3.

**Table 3:** Chronological Progression of Clinical Presentation, Diagnostic Evaluation, and Management. MS: Multiple sclerosis; NMDA: N-methyl-D-aspartate

Phase of Hospitalization	Key Findings	Diagnostic/Management Implications
Initial Presentation	Acute psychosis with behavioral changes, agitation, and altered mental status in a patient with known multiple sclerosis and no prior psychiatric history.	Differential diagnosis included primary psychiatric illness, atypical MS relapse, infectious encephalitis, substance-related causes, and autoimmune encephalitis.
Neurologic Progression	Development of focal seizures with worsening encephalopathy.	Clinical progression beyond the typical spectrum of MS relapse increased concern for a primary central nervous system process. Neurology consultation obtained.
Cerebrospinal Fluid Evaluation	CSF demonstrated marked lymphocytic pleocytosis with normal glucose and protein levels; infectious studies were negative.	Findings supported a nonbacterial inflammatory process and lowered suspicion for infectious encephalitis.
Electroencephalographic Evaluation	EEG demonstrated focal seizure activity arising from the left frontal region. Subsequent recordings showed diffuse slowing with superimposed beta activity interpreted by the epilepsy service as resembling a delta brush–like pattern.	Findings supported seizures and severe encephalopathy and increased suspicion for anti-NMDA receptor encephalitis.
Autoimmune Evaluation	Anti-NMDA receptor antibody testing was sent because of progressive neuropsychiatric deterioration, seizures, inflammatory CSF findings, and nondiagnostic neuroimaging.	Autoimmune encephalitis became the leading diagnostic consideration.
Clinical Deterioration	Progression to catatonia, mutism, orofacial dyskinesias, and autonomic instability.	Development of characteristic features further strengthened the suspicion of anti-NMDA receptor encephalitis.
Empiric Immunotherapy	High-dose intravenous corticosteroids were initiated while antibody testing remained pending.	Early treatment was pursued because of high clinical suspicion and ongoing neurologic deterioration.
Escalation of Therapy	Plasma exchange (PLEX) was initiated and completed over five sessions.	First-line immunotherapy intensified because of severe disease and continued clinical decline.
Diagnostic Confirmation	Anti-NMDA receptor antibodies returned positive in CSF (1:10) and serum (1:40).	Diagnosis of anti-NMDA receptor encephalitis confirmed.
Current Clinical Status	The patient remains hospitalized in the Neurocritical Care Unit under multidisciplinary management.	Long-term neurologic outcome, rehabilitation progress, and recovery trajectory remain unavailable at the time of manuscript preparation.

A limitation of this report is the absence of long-term outcome data. At the time of manuscript preparation, the patient remained hospitalized in the Neurocritical Care Unit and continued to receive active treatment. As a result, information regarding functional recovery, cognitive outcome, seizure recurrence, discharge disposition, and long-term immunotherapy could not be assessed.

## Discussion

Background (history, epidemiology, and risk factors)

AE comprises a group of immune-mediated inflammatory disorders of the central nervous system characterized by rapidly progressive neuropsychiatric symptoms, including psychiatric disturbances, seizures, cognitive impairment, and altered consciousness [1,2]. Anti-NMDA receptor encephalitis is among the most common and best-characterized forms of AE and predominantly affects young women [1,9,10]. The disorder is mediated by immunoglobulin G antibodies directed against the GluN1 subunit of the NMDA receptor, resulting in receptor internalization and synaptic dysfunction [1,10].

Clinically, anti-NMDA receptor encephalitis often follows a characteristic progression from psychiatric symptoms to seizures, encephalopathy, movement disorders, and autonomic instability [2,10]. Early diagnosis remains challenging because neuroimaging is frequently normal or nonspecific, while psychiatric manifestations may initially mimic primary psychiatric illness or infectious encephalitis [2,10].

Risk factors include underlying neoplasms, particularly ovarian teratomas; however, a substantial proportion of cases are nonparaneoplastic and occur in the absence of an identifiable tumor [5,10,11]. Increasing evidence suggests that overlapping autoimmune neurologic syndromes may occur in some patients with pre-existing autoimmune disease; however, the mechanisms underlying these associations remain incompletely understood [4,6,12].

MS is one such immune-mediated disorder in which overlapping autoimmune neurologic syndromes have been increasingly recognized [6,12,13]. Although coexistence of MS and anti-NMDA receptor encephalitis remains uncommon, this overlap is clinically important because neuropsychiatric symptoms, inflammatory CSF findings, and nonspecific imaging abnormalities may be prematurely attributed to MS alone, potentially delaying recognition of a treatable AE [5,7,13]. This case highlights the importance of maintaining a broad differential diagnosis when clinical features evolve beyond the expected spectrum of MS relapse.

Pathology/pathophysiology

Anti-NMDA receptor encephalitis is mediated by immunoglobulin G antibodies directed against the GluN1 subunit of the NMDA receptor, resulting in receptor internalization and impaired glutamatergic neurotransmission [1,3]. Because NMDA receptors play a critical role in synaptic transmission, neuronal plasticity, learning, and memory, antibody-mediated dysfunction can produce a characteristic constellation of psychiatric symptoms, seizures, cognitive impairment, dyskinesias, catatonia, and autonomic instability [1-3]. Unlike many autoimmune neurologic disorders, anti-NMDA receptor encephalitis is largely non-cytotoxic, with limited neuronal destruction, which may contribute to its potential reversibility with timely immunotherapy [3,5].

MS is a chronic immune-mediated demyelinating disorder characterized by autoreactive T-cell and B-cell responses leading to inflammation, demyelination, and neuroaxonal injury [6,7]. Although MS may be associated with cognitive and psychiatric symptoms, acute psychosis, seizures, and rapidly progressive encephalopathy are uncommon manifestations of MS relapse [6,7].

The relationship between MS and anti-NMDA receptor encephalitis remains incompletely understood. Although overlapping autoimmune neurologic syndromes have been reported, a causal relationship has not been established, and their coexistence may reflect either shared immune mechanisms or coincidental occurrence [12,13]. In the present case, the patient's rapidly progressive evolution from psychosis to seizures, encephalopathy, dyskinesias, catatonia, and autonomic instability, together with inflammatory CSF findings, EEG abnormalities, and anti-NMDA receptor antibody positivity, was more consistent with anti-NMDA receptor encephalitis than isolated MS relapse. The principal clinical significance of the coexisting MS diagnosis was its potential to introduce diagnostic anchoring and complicate early recognition of a superimposed autoimmune encephalitic process.

Comparative analysis with current literature (clinical presentation, diagnostic workup, management, and outcome)

Clinical Presentation

The typical clinical course of anti-NMDA receptor encephalitis involves a characteristic progression from psychiatric symptoms to seizures, followed by encephalopathy, movement disorders, and autonomic instability [1,2,10]. In contrast, MS relapse more commonly presents with focal neurologic deficits such as motor weakness, sensory disturbances, or visual impairment, with acute psychosis and seizures being relatively uncommon features [4,6,7]. 

In this case, the patient’s presentation closely mirrored the classic trajectory of anti-NMDA receptor encephalitis, beginning with acute psychosis and rapidly progressing to focal seizure activity and severe encephalopathy [1,2]. The subsequent development of catatonia, orofacial dyskinesias, and autonomic instability further supported this diagnosis, as these features are well-described manifestations of advanced disease [2,10]. However, the presence of underlying MS introduced significant diagnostic complexity, as neuropsychiatric symptoms may occur in MS but are typically subacute and rarely exhibit this rapid progression [6,7]. The abrupt evolution of symptoms therefore served as a key distinguishing feature favoring AE over MS relapse [2,4].

Diagnostic Workup

Neuroimaging in anti-NMDA receptor encephalitis is frequently normal or demonstrates nonspecific findings, limiting its sensitivity in early disease [2,4]. In contrast, MS relapse is typically associated with new demyelinating lesions on MRI, often with contrast enhancement [6]. In this patient, the absence of new enhancing or demyelinating lesions argued against an active MS flare and supported an alternative diagnosis [5,6].

CSF analysis in AE commonly demonstrates lymphocytic pleocytosis with mild protein elevation, findings that are supportive but not specific [2,4]. The patient’s CSF profile, characterized by marked lymphocytic pleocytosis, was consistent with an inflammatory process and aligned with reported findings in AE [2,4]. Negative infectious testing further reduced the likelihood of infectious encephalitis, strengthening the suspicion for an autoimmune etiology [2,4].

EEG abnormalities are frequently observed in anti-NMDA receptor encephalitis and may include diffuse slowing, epileptiform activity, and characteristic patterns such as extreme delta brush [2]. In this case, initial EEG captured focal left frontal seizures, including focal unaware seizures and focal-to-bilateral tonic-clonic seizures, confirming active cortical involvement. Subsequent prolonged EEG monitoring demonstrated diffuse background slowing with superimposed beta activity interpreted by the epilepsy service as resembling a delta brush pattern, findings consistent with encephalopathy in the appropriate clinical context [2,10]. The evolution from focal seizure activity to diffuse encephalopathic abnormalities highlights the value of serial EEG assessment in patients with suspected AE and is consistent with previously reported electroencephalographic manifestations of anti-NMDA receptor encephalitis [2,10].

Management

Standard management of anti-NMDA receptor encephalitis includes early initiation of immunotherapy, such as corticosteroids, intravenous immunoglobulin, or plasma exchange, with escalation to second-line agents in refractory cases [2,6]. Early treatment has been consistently associated with improved neurologic outcomes and reduced long-term morbidity [2,5].

In this case, management followed a parallel approach, incorporating empiric antimicrobial therapy during the initial diagnostic phase while initiating immunotherapy based on clinical suspicion [2,4,5]. This strategy aligns with current recommendations emphasizing early treatment in suspected AE, even prior to confirmatory antibody results [2,5]. The coexistence of MS required careful consideration of immunosuppressive therapy, balancing the need for prompt treatment against the risk of infection and overlapping immune modulation [4,6].

Clinical Outcome

Prognosis in anti-NMDA receptor encephalitis is generally favorable with timely treatment, with approximately 75-80% of patients achieving substantial recovery [2,6]. However, delayed diagnosis and treatment are associated with worse neurologic outcomes, prolonged hospitalization, and increased morbidity [2,5]. Recurrence occurs in approximately 10-20% of cases, particularly in nonparaneoplastic disease or in patients who do not receive complete immunotherapy [5].

In this patient, the severity of presentation, including encephalopathy, dyskinesias, and autonomic instability, reflects features associated with more severe disease and prolonged recovery [2,5]. The presence of underlying MS may further complicate recovery due to baseline immune dysregulation, although data on overlapping autoimmune neurologic conditions remain limited [4,6]. This case underscores the importance of early recognition and treatment, particularly in patients with pre-existing autoimmune disease, where diagnostic uncertainty may delay appropriate management [2,6].

A summary of the distinguishing clinical, radiographic, and laboratory features between anti-NMDA receptor encephalitis and MS relapse is provided in Table 4, highlighting key findings that supported the final diagnosis.

**Table 4:** Summary of the Comparative Analysis With the Current Literature. This table compares the clinical, diagnostic, and imaging features of the present case with typical presentations of anti-NMDA receptor encephalitis and multiple sclerosis relapse. Citation numbers correspond to referenced literature supporting characteristic findings. The comparison highlights key distinguishing features, particularly the presence of acute psychosis, seizures, and inflammatory cerebrospinal fluid findings in the absence of new demyelinating lesions, supporting autoimmune encephalitis over multiple sclerosis relapse. NMDA: N-methyl-D-aspartate

Feature	Typical Anti-NMDA Receptor Encephalitis	Typical Multiple Sclerosis Relapse	Present Case
Age/Sex	Young women; median age early 20s [1,2]	Young adults with female predominance [6]	27-year-old female
Initial Presentation	Psychiatric symptoms, agitation, psychosis [1,2]	Focal neurologic deficits (motor, sensory, visual) [6,7]	Acute psychosis
Seizures	Common [1,2]	Uncommon [6,7]	Present (focal seizures)
Clinical Progression	Psychiatric symptoms → seizures → encephalopathy → dyskinesias/autonomic instability [2,10]	Gradual focal neurologic relapse [6]	Psychosis → seizures → encephalopathy → catatonia/dyskinesias
MRI Findings	Often normal or nonspecific [2,4]	New demyelinating lesions with possible enhancement [6]	No new demyelinating lesions;
CSF Findings	Lymphocytic pleocytosis with mild protein elevation [2,4]	Mild pleocytosis and oligoclonal bands [6]	Lymphocytic pleocytosis (145 WBC/µL)
EEG Findings	Diffuse slowing, epileptiform discharges, possible extreme delta brush [2,10]	Usually normal or nonspecific [6]	Focal left frontal seizures on initial EEG; subsequent prolonged EEG demonstrated diffuse slowing with superimposed beta activity suggestive of a delta brush pattern
Infectious Workup	Typically negative [2,4]	Not characteristic	Extensive infectious studies negative
Paraneoplastic Association	Ovarian teratoma in a subset of patients [11]	None	No neoplasm identified
Autoantibodies	Anti-GluN1 NMDA receptor antibodies [1,3]	No NMDA receptor antibodies	Positive CSF and serum anti-NMDA receptor antibodies
Response to Immunotherapy	Favorable with early treatment [2,5]	Often steroid responsive [6]	Treated with corticosteroids and plasma exchange

What we learned from this case 

This case highlights several important clinical lessons regarding the diagnosis and management of AE in patients with pre-existing neurologic disease.

First, clinicians should be cautious about attributing new neurologic symptoms solely to a known diagnosis of MS. Although MS may be associated with cognitive and psychiatric manifestations, the abrupt onset of psychosis followed by seizures, severe encephalopathy, catatonia, dyskinesias, and autonomic instability is atypical for MS relapse and should prompt consideration of alternative or superimposed pathologies. This case illustrates the risk of diagnostic anchoring, in which a pre-existing diagnosis may narrow the differential diagnosis and delay recognition of a separate disease process.

Second, normal or nonspecific neuroimaging does not exclude AE. In this patient, brain MRI demonstrated no active demyelination or other findings sufficient to explain the severity of the clinical presentation. Recognition of the characteristic progression from psychiatric symptoms to seizures and encephalopathy proved more informative than neuroimaging alone, underscoring the importance of clinical pattern recognition in suspected AE.

Third, the diagnosis of anti-NMDA receptor encephalitis relied on the integration of multiple diagnostic modalities. CSF analysis demonstrated an inflammatory process, EEG identified focal seizures and subsequent diffuse encephalopathic abnormalities, infectious studies were unrevealing, and anti-NMDA receptor antibodies were ultimately detected in both CSF and serum. This case highlights the complementary roles of CSF analysis, EEG, and antibody testing when neuroimaging is nondiagnostic.

Finally, this case reinforces the importance of early empiric immunotherapy in patients with a high clinical suspicion for AE. Because antibody testing may require days to weeks to result, treatment decisions often must be made before definitive confirmation is available. In this patient, the decision to initiate corticosteroids and plasma exchange was guided by the overall clinical picture and was not deferred pending antibody results.

Although the coexistence of MS and anti-NMDA receptor encephalitis in this patient is noteworthy, this case cannot establish a causal relationship between the two conditions. Rather, the principal clinical lesson is that the presence of a known neurologic disorder should not preclude consideration of alternative diagnoses when new symptoms evolve beyond the expected spectrum of disease.

Limitations

This case has several limitations. Detailed historical information regarding the patient's MS was unavailable because the patient was encephalopathic and later nonverbal throughout much of the hospitalization, while family members could provide only limited collateral information. Prior neurology records, disease-modifying therapy history, previous neuroimaging studies, and information regarding prior MS relapses were not available for review. Additionally, long-term clinical outcome data were unavailable because the patient remained hospitalized in the Neurocritical Care Unit at the time of manuscript preparation. Nevertheless, the diagnosis of anti-NMDA receptor encephalitis was strongly supported by the characteristic clinical progression, inflammatory CSF findings, EEG abnormalities, exclusion of alternative etiologies, and confirmatory anti-NMDA receptor antibody positivity in both serum and CSF.

## Conclusions

This case highlights the diagnostic challenges of acute neuropsychiatric deterioration in patients with pre-existing neurologic disease and illustrates the potential for diagnostic anchoring to delay recognition of alternative, treatable conditions. In this patient with known MS, the progression from psychosis to seizures, encephalopathy, catatonia, dyskinesias, and autonomic instability was ultimately found to be attributable to anti-NMDA receptor encephalitis rather than isolated MS relapse.

The case underscores several important clinical lessons. First, acute psychosis and seizures are atypical manifestations of MS relapse and should prompt consideration of alternative diagnoses. Second, normal or nonspecific neuroimaging does not exclude AE, making clinical pattern recognition essential. Third, CSF analysis, EEG, and antibody testing provide complementary diagnostic information when imaging is nondiagnostic. Finally, early empiric immunotherapy should be considered when clinical suspicion for AE is high, even before confirmatory antibody results become available. Ultimately, this case emphasizes the importance of maintaining a broad differential diagnosis, avoiding premature diagnostic closure, and recognizing the characteristic clinical progression of anti-NMDA receptor encephalitis to facilitate timely diagnosis and treatment.
